# Increased expression of miR-320b in blood plasma of patients in response to SARS-CoV-2 infection

**DOI:** 10.1038/s41598-024-64325-9

**Published:** 2024-06-14

**Authors:** Aline de Souza Nicoletti, Marília Berlofa Visacri, Carla Regina da Silva Correa da Ronda, Julia Tiemi Siguemoto, Carolini Motta Neri, Rafael Nogueira de Souza, Deise de Souza Ventura, Adriana Eguti, Lilian Ferreira de Souza Silva, Mauricio Wesley Perroud Junior, Keini Buosi, Mehrsa Jalalizadeh, Franciele Dionato, Luciana Dal Col, Cristiane Giacomelli, Patrícia Leme, Leonardo Oliveira Reis, Luiz Augusto dos Santos, Nelson Durán, Wagner José Fávaro, José Luiz da Costa, Carolina Dagli-Hernandez, Patricia Moriel, Eder de Carvalho Pincinato

**Affiliations:** 1 School of Medical Science, Universidade Estadual de Campinas (UNICAMP), Campinas, SP Brazil; 2 School of Pharmaceutical Science, Universidade de São Paulo (USP), São Paulo, SP Brazil; 3 Faculty of Pharmaceutical Science, Universidade Estadual de Campinas (UNICAMP), Campinas, SP Brazil; 4 Hospital Estadual de Sumaré Dr. Leandro Francheschini, Sumaré, SP Brazil; 5 School of Life Sciences, Pontifical Catholic University of Campinas (PUC-Campinas), Campinas, SP Brazil; 6 Hospital Municipal de Paulínia, Paulínia, SP Brazil

**Keywords:** microRNA, miRNA, Epigenetic, COVID-19, SARS-CoV-2, Genetics, Biomarkers

## Abstract

Coronavirus disease 2019 (COVID-19) is caused by Severe Acute Respiratory Syndrome Coronavirus 2 (SARS-CoV-2). Recent research has demonstrated how epigenetic mechanisms regulate the host–virus interactions in COVID-19. It has also shown that microRNAs (miRNAs) are one of the three fundamental mechanisms of the epigenetic regulation of gene expression and play an important role in viral infections. A pilot study published by our research group identified, through next-generation sequencing (NGS), that miR-4433b-5p, miR-320b, and miR-16–2-3p are differentially expressed between patients with COVID-19 and controls. Thus, the objectives of this study were to validate the expression of these miRNAs using quantitative real-time polymerase chain reaction (qRT-PCR) and to perform in silico analyses. Patients with COVID-19 (n = 90) and healthy volunteers (n = 40) were recruited. MiRNAs were extracted from plasma samples and validated using qRT-PCR. In addition, in silico analyses were performed using mirPath v.3 software. MiR-320b was the only miRNA upregulated in the case group com-pared to the control group. The in silico analyses indicated the role of miR-320b in the regulation of the *KITLG* gene and consequently in the inflammatory process. This study confirmed that miR-320b can distinguish patients with COVID-19 from control participants; however, further research is needed to determine whether this miRNA can be used as a target or a biomarker.

## Introduction

Coronavirus disease 2019 (COVID-19) is caused by Severe Acute Respiratory Syndrome Coronavirus 2 (SARS-CoV-2)^1^. The main form of disease transmission involves the inhalation of respiratory droplets containing the virus, and the most common symptoms include fever, fatigue, and dry cough^2^. Most patients develop mild symptoms; however, some may progress to severe critical illness (19% of cases), pneumonia, severe acute respiratory syndrome, multiple organ dysfunction, and death^3^. Although advanced age and the presence of comorbidities such as diabetes, heart disease, obesity, and immunosuppression contribute to increased disease severity^4^, recent research has demonstrated how epigenetic mechanisms regulate host–virus interactions and can be decisive in the course of infections such as COVID-19^5^. Epigenetic regulation of gene expression does not involve changes in the DNA sequence, and the function of noncoding RNAs (ncRNAs), such as microRNAs (miRNAs), is one of the fundamental mechanisms of epigenetic regulation^6^.

MiRNAs are small RNA molecules comprising approximately 22 noncoding nucleotides^7^, which mostly bind in a complementary mode to the 3′ untranslated region (3′-UTR) of the target messenger RNA (mRNA). Depending on the complementarity of this binding, they can mediate mRNA degradation or inhibit its transcription, thereby regulating protein synthesis^8^. Furthermore, circulating miRNAs play an important role in viral infections, either in cellular antiviral responses or viral replication and propagation^9^.

To identify dysregulated miRNAs that could serve as biomarkers for COVID-19, we performed a scoping review of the literature in which the main miRNAs identified were miR-21-5p, miR-146a, miR-126-3p, miR-144, and miR-155. Among these miRNAs, miR-21-5p, miR-144, and miR-155 appear to be diagnostic biomarkers, while miR-146a appears to be a biomarker for disease severity^10^. In a review published by Lin et al., 18 deregulated host miRNAs, their binding sites, and their functions were reported. Of these, miR-16 appears to bind to the BCL2 gene of the SARS-CoV-2 genome and regulates apoptosis, whereas the miR-320 family (upregulated) appears to bind to IL-6, IL-8, and IP10, which are involved in the inflammatory process^11^.

Furthermore, using next-generation sequencing (NGS), a pilot study published by our research group identified 18 plasma miRNAs that are differentially expressed between patients with COVID-19 and controls, including miR-4433b-5p, miR-320b, and miR-16-2-3p^12^. A matrix constructed for the analysis of miRNA–gene interactions indicated that miR-320b interacted with most of the 242 genes selected in the enrichment analyses^12^. Therefore, based on our aforementioned pilot study, the aims of the present study were to validate the expression of hsa-miR-4433b-5p, hsa-miR-320b, and hsa-miR-16-2-3p as biomarkers of infection by SARS-CoV-2 in the plasma of a larger cohort of participants using the quantitative real-time polymerase chain reaction (qRT-PCR) technique, and perform in silico analyses to better understand how dysregulation of the expression of these miRNAs could contribute to the pathogenesis of COVID-19.

## Results

### Participants

A total of 130 participants were included: 40 in the control group and 90 in the case group. Of these, five from the case group were excluded from the analyses because they had the spiked cel-miR-39-3p expression levels above or below two standard deviations. Therefore, a total of 85 patients with COVID-19 (45 with mild/moderate COVID-19 and 40 with severe/critical COVID-19) were included.

The demographic and clinical characteristics of all participants are shown in Table 1.

**Table 1:** Demographic and clinical data of included participants*.

Variable	Control group (N = 40)	Case group (N = 85)	p-value
Age (mean ± SD, Years)	44.50 ± 18.88	48.48 ± 17.17	0.1968^1^
Sex (N, %)			
Male	21 (33.9%)	41 (66.1%)	0.6564^2^
Female	19 (30.2%)	44 (69.8%)	
Total	40	85	
Ethnicity (N, %)			
Caucasian	37 (60.7%)	24 (39.3%)	** < 0.0001**^2^
Non-Caucasian	3 (13.0%)	20 (87.0%)	
Total	40	44**	
Comorbidities (N, %)			
No	26 (55.3%)	21 (44.7%)	** < 0.0001**^2^
Yes	14 (17.9%)	64 (82.1%)	
Total	40	85	
Diabetes (N, %)			
No	40 (47.1%)	45 (52.9%)	** < 0.0001**^2^
Yes	0 (0.0%)	26 (100.0%)	
Total	40	71**	
Systemic arterial hypertension (N, %)			
No	32 (45.1%)	39 (54.9%)	**0.0083**^2^
Yes	8 (20.0%)	32 (80.0%)	
Total	40	71**	
Ischemic heart disease (N, %)			
No	40 (37.4%)	67 (62.6%)	0.2946^3^
Yes	0 (0.0%)	4 (100.0%)	
Total	40	71**	
Chronic obstructive pulmonary disease (N, %)			
No	40 (37.0%)	68 (63.0%)	0.5519^3^
Yes	0 (0.0%)	3 (100.0%)	
Total	40	71**	

*SD* standard deviation, *N* sample number.

p-value less than 0.05 values are in bold.

^1^Based on Mann–Whitney test.

^2^Chi-squared test.

^3^Fisher’s exact test.

*The percentage was calculated horizontally, in which the sum of sample N between the control and case groups corresponds to 100%.

**Data not available for the entire sample.

Statistical analysis showed that the control and case groups did not differ with respect to age or sex; however, differences were observed in terms of ethnicity and the presence of comorbidities. Most participants in the control group self-identified as Caucasians (60.7%), whereas most of the case group self-identified as non-Caucasians (87.0%). Regarding the presence of comorbidities, the case group had more comorbidities (82.1%), such as diabetes (100%) and systemic arterial hypertension (80.0%), compared to the control group.

The demographic and clinical characteristics of patients with mild/moderate and severe/critical COVID-19 are shown in Supplementary Tables S1 and S2, respectively.

Regarding the use of medications during hospitalization and the outcome of the disease, of the 40 patients with severe–critical COVID-19, 31 (77.5%) received 6 mg of dexamethasone once a day, 24 (60%) received 75 mg of oseltamivir twice a day, and 18 (45%) died.

Regarding vaccination against COVID-19, until blood collection was carried out, no patient with severe/critical COVID-19 was vaccinated. Of the 45 included patients with mild/moderate COVID-19, 22 (48.9%) were unvaccinated and of the 40 control subjects, 17 (42.5%) were also unvaccinated. No analysis between the “vaccinated” and “unvaccinated” subgroups was performed.

### Validation of miRNAs via qRT-PCR

**Figure 1:**
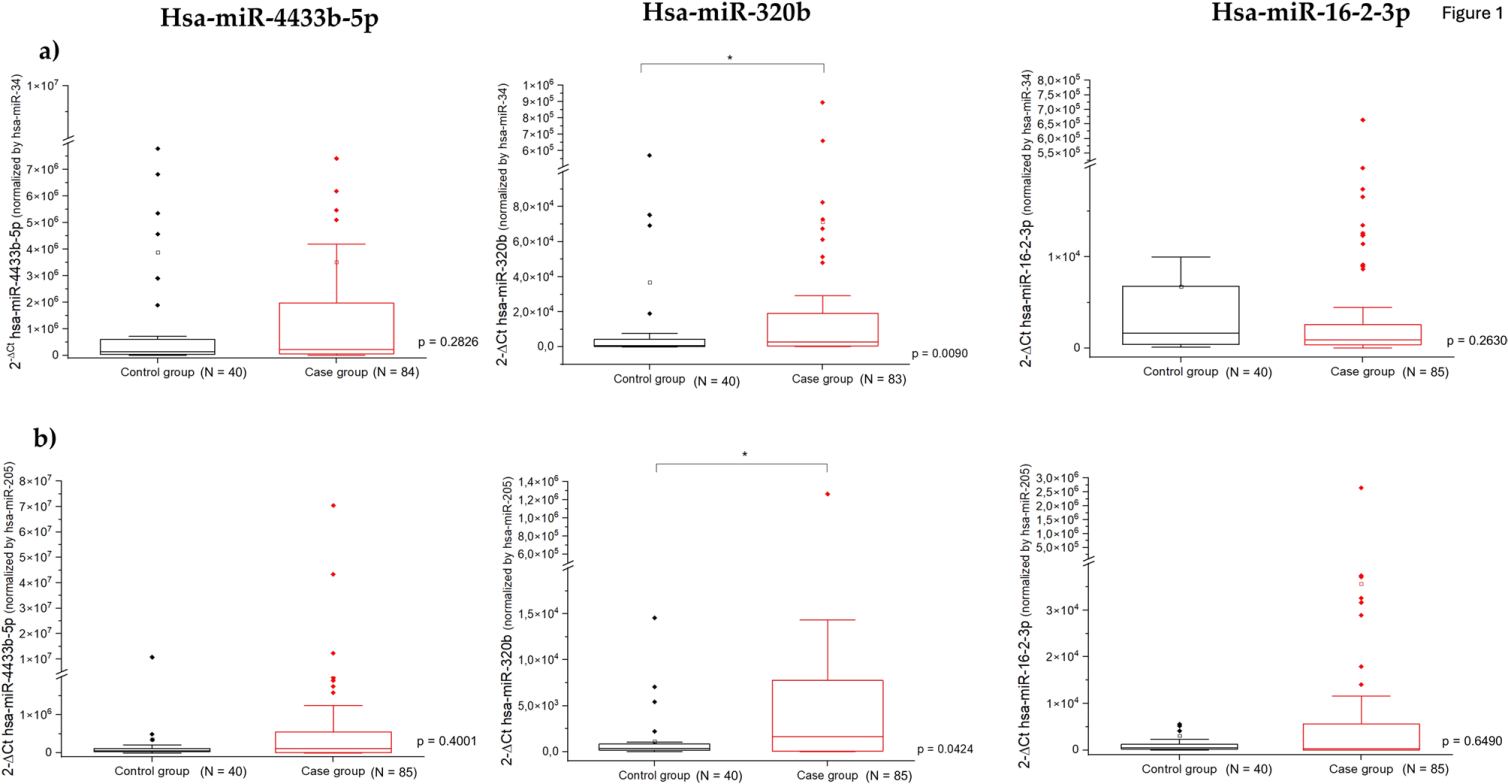
Gene expression of plasmatic miR-4433b-5p, miR-320b, and miR-16–2-3p between the control and case groups; (**a**) miR-NAs normalized to endogenous control hsa-miR-34a-3p and (**b**) miRNAs normalized to endogenous control hsa-miR-205-3p. The p-value and sample number (N) for the case and control groups are described for each comparison.

Figure 1 presents the gene expression of the three validated plasmatic miRNAs between control and case groups that was analyzed using the 2^−ΔCt^ method. For miR-4433b-5p, 1 patient in the Case group was removed from the analyzes as it was not possible to normalize by miR-34a -3p, while for miR-320b, 2 patients were removed from the analysis, as either it was not possible to normalize by miR-34a-3p or the sample did not amplify in the RT-qPCR.

**Figure 2:**
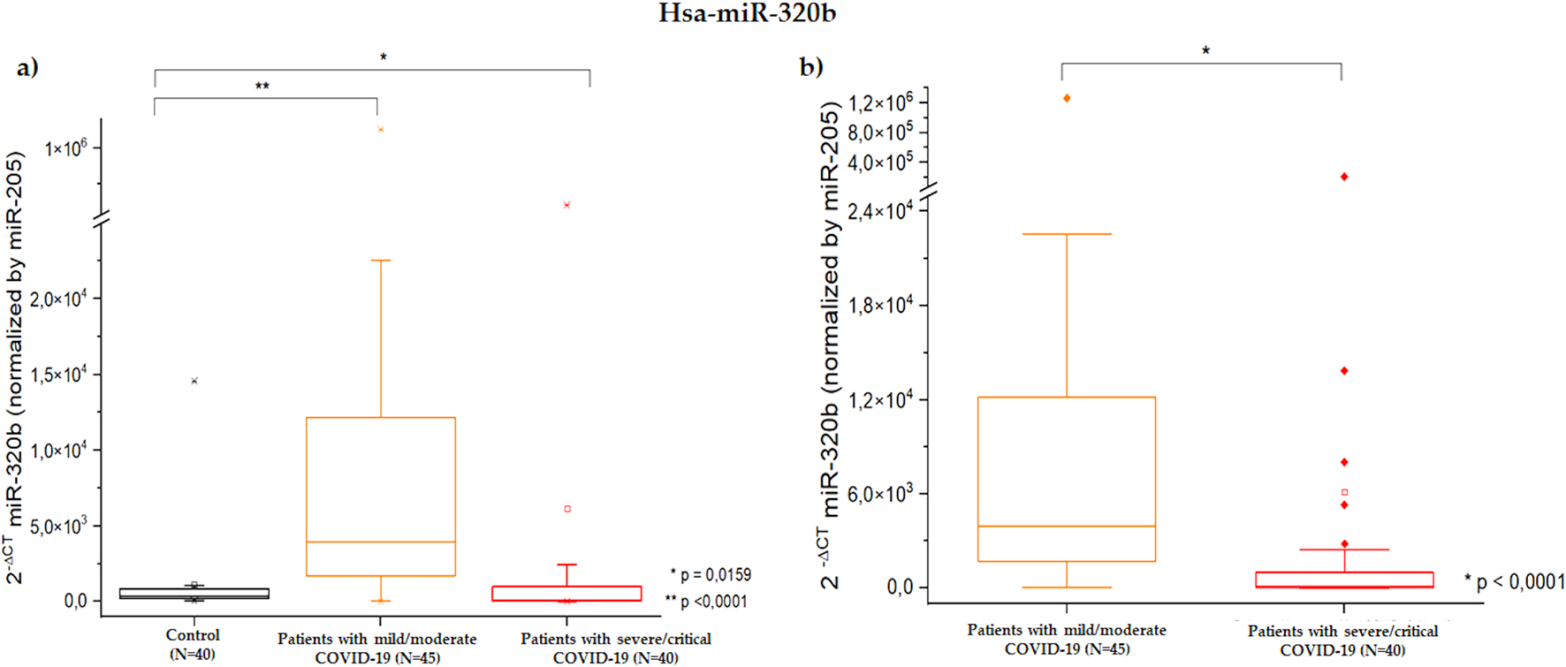
Gene expression of plasmatic miR-320b normalized to endogenous control hsa-miR-205-3p. (**a**) Comparison between controls, patients with mild/moderate COVID-19, and patients with severe/critical COVID-19. (**b**) Comparison between patients with mild/moderate COVID-19 and patients with severe/critical COVID-19. The p-value and sample number (N) for the case and control groups are described for each comparison.

Among these, miR-320b was the only miRNA whose expression was significantly upregulated in the case group compared to the control group, regardless of the endogenous normalizer used. More specifically, miR-320b, normalized to miR-205-3p, was more highly expressed in both subgroups of patients with COVID-19 than in the control group (patients with mild/moderate COVID-19, p < 0.0001; patients with severe/critical COVID-19, p = 0.0159) (Fig. 2a); however, patients with severe/critical COVID-19 ((2^−ΔCt^ = 6.13 × 10^3^ ± 32.00 × 10^3^) showed less upregulated expression of miR-320b compared to patients with mild/moderate COVID-19 (2^−ΔCt^ = 36.17 × 10^3^ ± 187.52 × 10^3^; p < 0.0001), when normalized to miR-205-3p (Fig. 2b).

When we compared the expression of miR-320b, normalized to miR-205-3p, with the outcome of death in patients with severe/critical COVID-19, we observed that lower expression of miR-320b was related to death (deceased: 2^−ΔCt^ = 0.80 × 10^3^ ± 1.89 × 10^3^; recovered: 2^−ΔCt^ = 11.00 × 10^3^ ± 44.10 × 10^3^; p = 0.0422).

For miR-4433b-5p and miR-16–2-3p, no significant differences were found. The fold regulation (FR) and p-values for each validated miRNA are shown in Supplementary Table S3, according to the endogenous normalizer used.

### ROC curves

**Figure 3:**
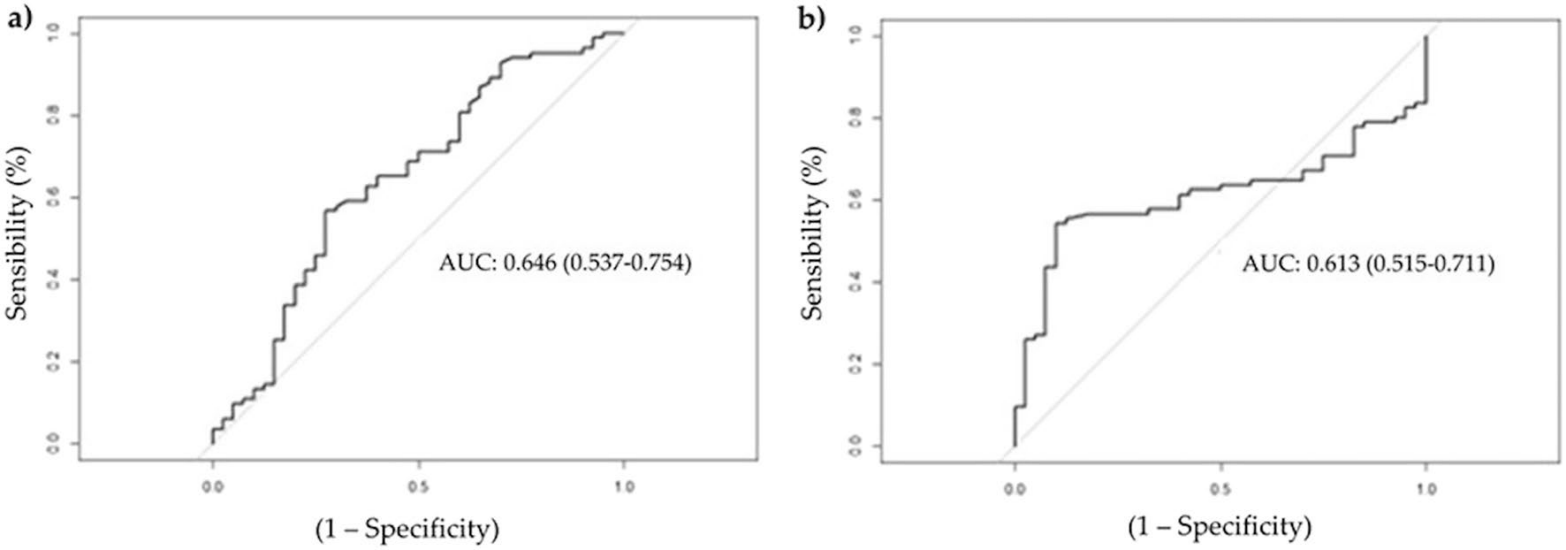
ROC curves of miR-320b expression between the control and case groups. The ordinate represents the sensitivity values, expressed as a percentage, and the abscissa represents the specificity values; (**a**) miR-320b normalized by endogenous control hsa-miR-34a-3p and (**b**) miR-320b normalized by endogenous control hsa-miR-205-3p; AUC: Area under curve.

For miR-320b (the only miRNA for which a significant difference in gene expression was observed between the evaluated groups), an ROC curve was generated to assess its ability to distinguish between participants without COVID-19 and patients with COVID-19 (Fig. 3).

Table 2 presents the AUC, cut-off point, sensitivity, specificity, PPV, and NPV values of miR-320b between the control and case groups according to the normalizer used.

**Table 2:** AUC, cutoff point, sensitivity, specificity, positive predictive value, and negative predictive value of miR-320b between the control and case groups, control and patients with mild/moderate COVID-19, and control and patients with severe/critical COVID-19.

Groups	Normalizer	Area	Cutoff	Sensitivity	Specificity	PPV	NPV
Control versus case	miR-34a-3p	0.646	1887	0.566	0.725	0.810	0.446
	miR-205-3p	0.613	1194	0.541	0.900	0.920	0.480
Control versus patients with mild/moderate COVID-19	miR-34a-3p	0.556	–	–	–	–	–
	miR-205-3p	0.853	1272	0.800	0.900	0.900	0.800
Control versus patients with severe/critical COVID-19	miR-34a-3p	0.747	1894	0.821	0.725	0.744	0.806
	miR-205-3p	0.657	120	0.575	0.825	0.767	0.660

*PPV* positive predictive value, *NPV* negative predictive value.

Reasonable AUC and sensitivity values were observed for both normalizers, except for the miR-34a-3p normalizer, in the comparison between the control group and patients with mild/moderate COVID-19, where the AUC value was less than 0.6 and, therefore, the other parameters were not calculated. Regarding the specificity and PPV parameters, desirable values of 72.5% and 81% were observed, respectively, when miR-320b expression was normalized to that of miR-34. These values increased to 90% and 92%, respectively, when the expression of miR-320b was normalized to that of miR-205. This finding indicates that this miRNA demonstrates good specificity; that is, it is useful for identifying truly negative patients. When these parameters were compared between the control group and patients with mild/moderate COVID-19, the sensitivity and specificity of miR-320b expression normalized to miR-205-3p were 80% and 90%, respectively, and 57.5% and 82.5%, respectively, in the subgroup of control versus patients with severe/critical COVID-19.

### In silico analysis of potential targets of miR-320b

Using mirPath v.3 software and the TargetScan web server, 21 genes related to hsa-miR-320b were identified (Supplementary Table S4). In addition, five predicted target pathways regulated by hsa-miR-320b were identified, all with p-values < 0.05, and these are listed as follows: (1) hematopoietic cell lineage, (2) chemical carcinogenesis, (3) caffeine metabolism, (4) metabolism of xenobiotics by cytochrome P450, and (5) linoleic acid metabolism. Of these, the hematopoietic cell lineage and linoleic acid metabolism pathways may be related to COVID-19.

## Discussion

Regarding the demographic and clinical data of the included participants, age and sex were intentionally matched between the case and control groups, which was not possible for ethnicity or presence of comorbidities. The control group comprised significantly more non-Caucasian individuals when compared to the case group. In addition, the case group had a higher proportion of patients with diabetes and hypertension than the control group. Although these differences between groups may contribute to possible bias, they corroborate the findings of previous literature reviews^13,14^. For instance, a study conducted in China reported that in an initial cohort of 1590 patients with COVID-19, 399 (25.1%) had at least one comorbidity, whereas 130 (8.2%) had two or more comorbidities. Hypertension (16.9%), diabetes (8.2%), cardiovascular disease (3.7%), and chronic kidney disease (1.3%) were the most common conditions in all patients with COVID-19^15^. The American Heart Association’s COVID-19 cardiovascular disease registry study reported that Hispanic and Black patients accounted for more than half of the hospitalized COVID-19 patients, and consequently, more than half of all hospital deaths. This indicates that these populations are more likely to be hospitalized with a SARS-CoV-2 infection, and therefore carry a disproportionate burden of COVID-19 mortality.

Regarding validation of the selected miRNAs, while NGS revealed that miR-4433b-5p was downregulated in patients with COVID-19^12^, in the present study, no significant differences were found in the gene expression of this miRNA between the case and control groups for both selected endogenous normalizers. In contrast, in a study published by Giannella et al., analysis of small RNA sequencing data identified the downregulation of miR-4433b-5p in the serum of patients with COVID-19 compared to that in the serum of healthy controls. In addition, survival curve analysis confirmed that high leukocyte counts (> 9 × 10^9^/L) and low serum miR-4433b-5p levels upon admission were associated with increased mortality^16^.

Najafipour et al. employed a small RNA deep sequencing approach to screen for differentially expressed miRNAs in blood samples derived from patients with COVID-19, and miR-16-2-3p was among the top ten most upregulated miRNAs^13^. Similarly, Li et al. revealed that miR-16-2-3p was the most upregulated miRNA, with an FC of 1.6 compared to that in the control group^17^. Although this miRNA was the second most downregulated miRNA in a preliminary study published by our research group^12^, in the present study, the expression of miR-16-2-3p was not significantly different between the case and control groups, independent of the endogenous normalizer used.

Corroborating the findings of our preliminary study, we found that miR-320b was significantly upregulated in patients with COVID-19 compared to healthy volunteers. Despite the modest sensitivity values, both endogenous normalizers showed desirable values for specificity, as indicated by the ROC curve. This indicates that this miRNA is not upregulated in samples from individuals who are not infected with SARS-CoV-2.

This desirable specificity is useful for truly identifying patients without COVID-19, and 92% of the patients with upregulated miR-320b expression had COVID-19 (positive predictive value).

The sensitivity parameter showed that 54.1% of patients with COVID-19 showed upregulation of miR-320b and the rest were false negatives. This modest sensitivity value can be explained by the influence of the subgroup of severe/critical patients, in which miR-320b showed low sensitivity when normalized to miR-205-3p. When miR-320b was normalized to miR-34a-3p, it was not possible to construct the ROC curve for the subgroup comprising mild/moderate patients, therefore we focused on the results normalized to miR-205-3p to examine the proposed influence of miR-320b on COVID-19.

MiR-320b, normalized to miR-205-3p, was more expressed in both subgroups of patients with COVID-19 compared to the control group (patients with mild/moderate COVID-19, p < 0.0001; patients with severe/critical COVID-19, p = 0.0159). However, patients with severe/critical COVID-19 showed less upregulated expression of miR-320b compared to patients with mild/moderate COVID-19 when normalized to miR-205-3p, which contributed to a decreased sensitivity when the subgroups were combined. This comparison could indicate an important role for miR-320b in allowing the monitoring of disease progression in patients infected with SARS-CoV-2, helping to anticipate the prognosis, and choosing the best treatment strategy^18^. When we compared the expression of miR-320b, normalized to miR-205-3p, with the outcome of death in patients with severe/critical COVID-19, we observed that lower expression of miR-320b was related to death. Therefore, in the present study, higher miR-320b expression appeared to be protective against more severe forms and fatal outcomes.

Consistent with our results, a study published by Giannella et al. also identified that the expression of the miR-320 family (320a-3p, 320b, 320c, and 320d) was upregulated in patients with COVID-19 compared to healthy controls. Furthermore, ROC curve analysis revealed that high expression levels of the miR-320 family (except miR-320a-3p) showed > 90% sensitivity and specificity in discriminating between these two groups, regardless of disease severity. However, network analysis of the genes targeted by miRNAs showed that upregulation of the miR-320 family was associated with the targeting of several genes involved in antiviral defense, such as genes that encode cytokines, chemokines, and cytokine receptors (IFNL1, CCL5, IL2RB); C-reactive protein (CRP); ferritin light chain (FTL); cytochrome c (CYCS); matrix metalloproteinase 2 (MMP2); and proteins involved in intracellular trafficking (ARF1, DTCN5, SEC24A, SLC26A2)^16^.

Using NGS, Duecker et al. revealed that the expression of the miR-320 family was significantly downregulated in patients with severe respiratory failure induced by SARS-CoV-2 compared to that in the controls, mainly during the subsequent phase of the disease (< 7 days). Additionally, the expression of all miR-320 family members was significantly correlated with C-reactive protein, D-dimer, and IL-6 levels in the blood of patients with COVID-19 and healthy controls. Enrichment analysis was performed to identify the contributions of miR-320a, miR-320b, and miR-320c targets to biological processes, highlighting the role of pathways related to inflammation and endothelial dysfunction^19^. In contrast, Giuliani et al. validated the overexpression of miR-320b in the serum of deceased patients using qRT-PCR compared to that in the serum of those who survived COVID-19. Furthermore, Kaplan–Meier and Cox regression models confirmed that patients with 20% higher serum levels of miR-320b had a three-fold increased risk of death during hospitalization due to COVID-19^20^.

**Figure 4:**
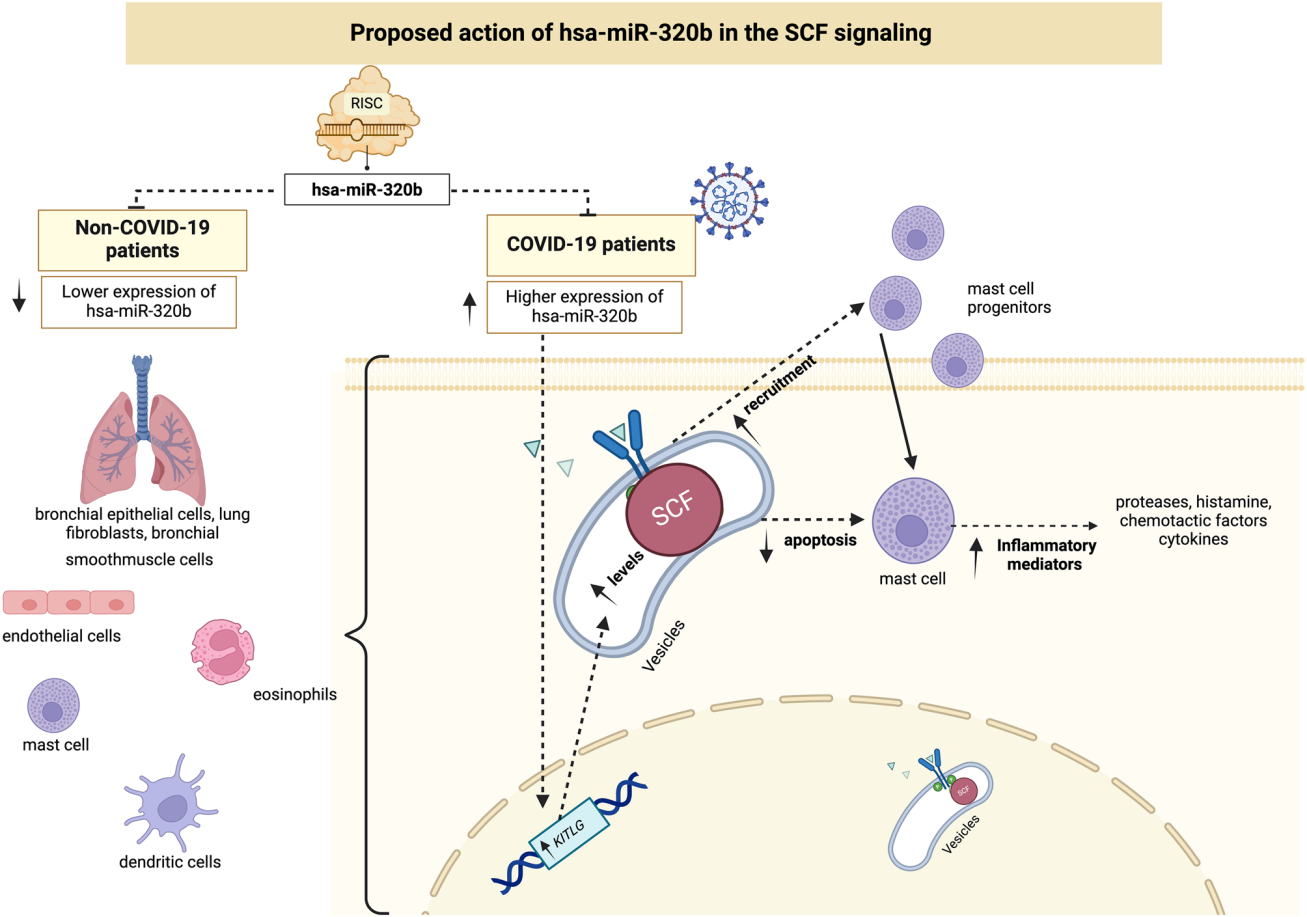
Proposed action of has-miR-320b in the SCF pathway. Increased hsa-miR-320b expression in patients with COVID-19 leads to an increase in KITLG gene expression and SCF protein. As a result, there is an increase in the recruitment of mast pro-genitor cells, contributing to an increase in the release of inflammatory mediators, a characteristic feature of SARS-CoV-2 infec-tion. Created with BioRender.com, accessed on 01 October 2023.

In our study, the in silico analysis focused on hsa-miR-320b because, as shown above, among the miRNAs validated via qRT-PCR, it was the only miRNA with statistically significant differences in expression between individuals without COVID-19 and those affected by the disease. Using mirPath v3 software, we identified five signaling pathways potentially related to hsa-miR-320b. Among these, the hematopoietic cell lineage and linoleic acid metabolism pathways were most likely to be involved in the pathogenesis of COVID-19. Among the genes associated with the hematopoietic cell lineage pathway, *KITLG* seems to encode a protein, the stem cell factor (SCF), whose increased expression appears to be related to the severity of the disease induced by SARS-CoV-2^21^. In Fig. 4, we propose a mechanism of action of hsa-miR-320b based on its relationship with the hematopoietic cell lineage pathway. Increased expression of hsa-miR-320b in patients with COVID-19 leads to increased expression of the *KITLG* gene and CSF protein in bronchial epithelial and smooth muscle cells, lung fibroblasts, endothelial cells, mast cells, eosinophils, and dendritic cells. This results in heightened recruitment of mast cell progenitors and diminished mast cell apoptosis, culminating in escalated release of inflammatory agents like proteases, histamines, chemotactic factors, and cytokines. These factors are acknowledged to be elevated in COVID-19, particularly in its severe manifestation^22,23^.

While widespread vaccination has exhibited considerable potential in curtailing the spread of COVID-19 and subsequently decreasing mortality rates^24^, a significant question remains: how can we discern, among variables such as age and comorbidities, individuals who will advance to the critical stage of COVID-19? This identification is crucial for the accurate management and prevention of unfavorable outcomes^25^. Therefore, analysis of the miRNA profile of patients with COVID-19, such as that performed in this study, may help us better understand the evolution of the disease by identifying the signaling pathways regulated by these miRNAs, allowing the design of new therapeutic targets or drug repositioning^13^.

This study has two limitations: (1) For a better study design, the control and case groups should only differ in relation to COVID-19, to avoid possible biases in the interpretation of the results. In our study, although we matched our groups regarding sex and age, the same was not possible regarding ethnicity and comorbidities such as diabetes and systemic arterial hypertension. (2) Only an in silico analysis of the pathways and genes involved with hsa-miR-320b was performed. The proposal for the signaling pathways was made based on software; however, carrying out laboratory analyzes to verify the pathway proposed in this article based only on in silico analysis would be ideal.

In conclusion, this study has confirmed that miR-320b distinguishes patients infected with SARS-CoV-2 from control participants, and that its higher expression in patients with COVID-19 may regulate the inflammatory process. Additionally, miR-320b expression is higher in patients with mild/moderate COVID-19 than in those with severe/critical variants. Furthermore, when we compared the expression of miR-320b with the outcome of death in patients with severe/critical COVID-19, we observed that lower expression of miR-320b was related to death, suggesting that lower expression of this miRNA may be related to greater severity of COVID-19. However, further research is required to determine whether this miRNA can be used as a severity biomarker for COVID-19.

## Materials and methods

### Study design and ethical considerations

This was an observational, analytical, case–control study conducted using non-probabilistic sampling. The study was conducted in accordance with the declaration of Helsinki and approved by the ethics committee of the Universidade Estadual de Campinas (UNICAMP) (numbers 36041420.0.000.5404 and 31049320.7.1001.5404). All participants or their guardians signed a consent form authorizing the use of their samples and data.

### Participants and eligibility criteria

Patients with COVID-19 (case group) were recruited from the Hospital Estadual Sumaré Dr. Leandro Francheschini (HES) in the city of Sumaré (SP, Brazil); the outpatient clinic of the community health center (CeCom) of the Universidade Estadual de Campinas, UNICAMP (Campinas-SP, Brazil); and Paulínia Municipal Hospital, HMP (Paulínia-SP, Brazil). Healthy volunteers (control group) were recruited from the UNICAMP community. The case group included patients with mild/moderate and severe/critical COVID-19 who were classified according to the National Institutes of Health (NIH) severity criteria^26^.

Eligibility criteria were as follows: age ≥ 18 years and admission to HES, CeCom, or HMP after confirmation through a nasopharyngeal swab of a positive RT-PCR result for SARS-CoV-2 for patients with COVID-19; and age ≥ 18 years and negative RT-PCR result for SARS-CoV-2 for control participants. Exclusion criteria for the control group were pregnancy, physical trauma, and/or immunological/hematological diseases.

### Characterization of participants

Participants were characterized in terms of age, sex, ethnicity, and comorbidities, such as diabetes, systemic arterial hypertension, ischemic heart disease, and chronic obstructive pulmonary disease.

Patients in the group with severe–critical COVID-19 were also characterized in relation to the use of medications during hospitalization and the outcome of the disease (recovered/death).

### Sample collection

Blood was collected in tubes containing ethynediaminetetraacetic acid (EDTA) as an anticoagulant and centrifuged at 2500 rpm for 10 min at 4 ℃ to separate the plasma. The plasma was aliquoted and stored in a freezer at − 80 ℃ until RNA extraction was performed. The average times between the onset of symptoms and blood collection for patients in the severe–critical COVID-19 group and for patients in the mild–moderate COVID-19 group were 8.33 days and 13.15 days, respectively. For patients in the severe–critical group, both blood collection and severity assessment occurred at the time of hospital admission.

The sample collection period for patients in the case group was from May 2020 to May 2022. According to the Oswaldo Cruz Foundation (FIOCRUZ), the predominant COVID-19 variants during this period were B.1.1.28, B.1.1.33, P.2, Alpha, and Gamma^27^. For participants in the control group, the collection period was from August 2020 to May 2022.

All analyses were performed at the Clinical Pharmacy Laboratory (CLIPHAR) located in the Faculty of Pharmaceutical Sciences at UNICAMP.

### Criteria for selecting possible miRNA candidates

Considering the RNA-Seq expression data obtained in a preliminary study by our research group^12^, three criteria were used to select the miRNAs for validation via qRT-PCR: (1) fold-change (FC) > 1.6 or <− 1.6, (2) p-value < 0.05, and (3) no comments from the GeneGlobe Data Analysis Center (Qiagen; https://geneglobe.qiagen.com/us/analyze). The FC results calculated via GeneGlobe analysis may be accompanied by type A, B, or C comments. In type A comments, the expression of the evaluated miRNA is relatively low in the control group or test group and reasonably high in the other group, which suggests that the actual FC value is at least as large as the calculated and reported FC result. In type B comments, the expression of the evaluated miRNA is relatively low in both the control group and the test group, suggesting that the FC result may have greater variations; therefore, for these two types of comments it is important to have a sufficient number of biological test replications to validate the result of the evaluated miRNA. In type C comments, the expression of the evaluated miRNA is equal to zero in the control and test groups, which means that its expression was not detected, making the FC result erroneous and uninterpretable. Therefore, the third criterion used to select the miRNAs for validation via qRT-PCR was the absence of any comments in the GeneGlobe analysis. Based on these criteria, three miRNAs were selected for validation using qRT-PCR, as described in the objectives of this paper: hsa-miR-4433b-5p, hsa-miR-320b, and hsa-miR-16–2-3p.

To select miRNA candidates for endogenous normalization, the following criteria were used: (1) FC equal to 1, (2) p-value equal to 0.999, and (3) no comments from the GeneGlobe Data Analysis Center. Normalization analysis was performed using the RefFinder online tool (http://blooge.cn/RefFinder/)^28–31^, which comprises four different normalization tools: BestKeeper, comparative DeltaCt, NormFinder, and GeNorm. The tools calculate a stability value for each candidate gene; the lower the stability value, the more stable the expression of the gene. Furthermore, the differences in mean ΔCt values (candidate Ct miR – Ct Cel-miR-39) for the case and control groups were analyzed for each miRNA candidate using a non-paired Student's t-test for the consideration of endogenous normalizers. The selected miRNAs should not have had their expression altered, regardless of the group/treatment in which they were allocated. Based on these criteria, hsa-miR-34a-3p and hsa-miR-205-3p were selected as endogenous controls.

### Assessment of miRNA expression

miRNA was extracted using the miRNeasy Serum/Plasma kit (Qiagen, Germany) following the manufacturer’s instructions. Immediately after the addition of QIAzol, 15 fmol of the exogenous control (spike-in) cel-miR-39 (Integrated DNA Technologies, IDT) was added to monitor the quality of processing and quantification of the samples. cDNA was synthesized using the TaqMan™ Advanced miRNA cDNA Synthesis Kit (Applied Biosystems, Waltham, MA, USA) following the manufacturer's instructions.

qRT-PCR reactions were performed on the Rotor-Gene Q platform (Qiagen) using TaqMan™ Advanced miRNA Assays (Applied Biosystems) for the three miRNAs selected for validation, as well as for cel-miR-39 (spike-in), hsa-miR-34a-3p, and hsa-miR-205-3p (endogenous normalizers). The total reaction volume was reduced to 10 μL, comprising 5 μL of TaqMan® Fast Advanced Master Mix (2 ×) (Applied Biosystems), 0.5 μL of TaqMan® Advanced miRNA Assay (20 ×) (Applied Biosystems), 2 μL of RNase-free water, and 2.5 μL of diluted cDNA (1:10). All reactions were performed in duplicate, and the cycling conditions were as follows: Hold: 95 °C, 20 s; Cycling: 95 °C, 15 s; 60 °C, 60 s. Raw data were evaluated using Rotor-Gene Q Series software (Qiagen). As a quality control measure, samples with cel-miR-39 expression above two standard deviations were excluded from the analysis.

Expression changes were evaluated using the 2^−ΔCt^ method, where ΔCt reflects the difference between the threshold cycle of the target gene and the endogenous control (hsa-miR-34a-3p and has-miR-205-3p). This step was performed to remove variations that were unrelated to the studied biological conditions.

### In silico analysis of has-miR-320b

As hsa-miR-320b was the only validated miRNA that distinguished between participants with and without COVID-19, in silico analysis was performed to identify the predicted target pathways for this miRNA related to the pathogenesis of SARS-CoV-2 infection. The analysis was performed using the online software mirPath v.3, based on the TargetScan web server (https://dianalab.e-ce.uth.gr/html/mirpathv3/index.php?r=mirpath#mirnas=hsa-miR-320b&methods=TargetScan&selection=0, accessed on August 1, 2023).

### Statistical analysis

To describe the sample profile, frequency tables of categorical variables were created using absolute frequencies (n), percentages (%), and descriptive measures (mean and standard deviation). For comparisons between groups, chi-squared (when the number of participants isn’t too small) or Fisher’s exact tests were used for categorical variables and the Mann–Whitney test was used for numeric variables. Receiver operating characteristic (ROC) curves were constructed using diagnostic measures such as area under curve (AUC), cut-off point, sensitivity, specificity, positive predictive value (PPV), and negative predictive value (NPV). The significance level was set at 5%.

### Institutional review board statement

The study was conducted in accordance with the Declaration of Helsinki and approved by the Ethics Committee of the Universidade Estadual de Campinas (protocol codes: 36,041,420.0.000.5404, August 15, 2020, and 31,049,320.7.1001.5404, July 25, 2020).

### Informed consent statement

Informed consent was obtained from all the subjects involved in the study.

### Supplementary Information

Supplementary Information.

## Data Availability

The datasets generated and/or analyzed during the current study are available from the Research Data Repository of the University of Campinas, 10.25824/redu/WWUNT1.
