# Supplementary material for: Increased expression of miR-320b in blood plasma of patients in response to SARS-CoV-2 infection
Source: Sci Rep. 2024 Jun 14;14:13702. doi: 10.1038/s41598-024-64325-9 (PMC11176351; doi:10.1038/s41598-024-64325-9)
Supplement: Supplementary file 1 — Supplementary Information. [file 41598_2024_64325_MOESM1_ESM.docx]

| **Variable**  **Table S1:** Demographic and clinical characteristics of control participants versus patients with mild/moderate COVID-19 | **Control group (N = 40)** | **Mild/moderate COVID-19 (N = 45)** | **p-value** |
| --- | --- | --- | --- |
| **Age (mean ± SD, Years)** | 44.50 ± 15.88 | 43.00 ± 11.75 | 0.6660^1^ |
| **Gender (N, %)** |  |  |  |
| Male | 21 (46.7%) | 24 (53.3%) | 0.9388^2^ |
| Female | 19 (47.5%) | 21 (52.5%) |  |
| Total | 40 | 85 |  |
| **Ethnicity (N, %)** |  |  |  |
| Caucasian | 37 (92.5%) | 3 (7.5%) | 0.3268 |
| Non-Caucasian | 3 (75.0%) | 1 (25.0%) |  |
| Total | 40 | 4 |  |
| **Comorbidities (N, %)** |  |  |  |
| No | **26 (65.0%)** | **14 (35.0%)** | **0.0018**^2^ |
| Yes | **14 (31.1%)** | **31 (68.9%)** |  |
| Total | **40** | **45** |  |
| **Diabetes (N, %)** |  |  |  |
| No | **40 (59.7%)** | **37 (40.30%)** | **0.0144**^2^ |
| Yes | **0 (0.0%)** | **5 (100.0%)** |  |
| Total | **40** | **32** |  |
| **Systemic arterial hypertension (N, %)** |  |  |  |
| No | 32 (61.5%) | 20 (38.5%) | 0.0995^2^ |
| Yes | 8 (40.0%) | 12 (60.0%) |  |
| Total | 40 | 32 |  |
| **Ischemic heart disease (N, %)** |  |  |  |
| No | 40 (55.6%) | 32 (44.4%) | - |
| Yes | 0 (0.0%) | 0 (0.0%) |  |
| Total | 40 | 32 |  |
| **Chronic obstructive pulmonary disease (N, %)** |  |  |  |
| No | 40 (55.6%) | 32 (44.4%) | - |
| Yes | 0 (0.0%) | 0 (0.0%) |  |
| Total | 40 | 32 |  |

SD, standard deviation; N, sample number; ^1^: based on Mann-Whitney test; ^2^, Chi-square test; ^3^, Fisher's exact test.

| **Variable**  **Table S2:** Demographic and clinical characteristics of control participants versus patients with severe/critical COVID-19 | **Control group (N = 40)** | **Severe/critical COVID-19 (N = 40)** | **p-value** |
| --- | --- | --- | --- |
| **Age (mean ± SD, Years)** | **44.50 ± 15.88** | **54.65 ± 14.24** | **0.0047^1^** |
| **Gender (N, %)** |  |  |  |
| Male | 21 (55.3%) | 17 (44.7%) | 0.3705^2^ |
| Female | 19 (45.2%) | 23 (54.8%) |  |
| Total | 40 | 40 |  |
| **Ethnicity (N, %)** |  |  |  |
| Caucasian | **37 (63.8%)** | **21 (36.2%)** | **<0.0001^2^** |
| Non-Caucasian | **3 (13.6%)** | **19 (86.4%)** |  |
| Total | **40** | **40** |  |
| **Comorbidities (N, %)** |  |  |  |
| No | **26 (78.8%)** | **7 (21.2%)** | **<0.0001**^2^ |
| Yes | **14 (29.8%)** | **33 (70.2%)** |  |
| Total | **40** | **40** |  |
| **Diabetes (N, %)** |  |  |  |
| No | **40 (69.0%)** | **18 (40.30%)** | **<0.0001**^2^ |
| Yes | **0 (0.0%)** | **21 (100.0%)** |  |
| Total | **40** | **39** |  |
| **Systemic arterial hypertension (N, %)** |  |  |  |
| No | **32 (62.7%)** | **19 (37.3%)** | **0.0037^2^** |
| Yes | **8 (28.6%)** | **20 (71.4%)** |  |
| Total | **40** | **39** |  |
| **Ischemic heart disease (N, %)** |  |  |  |
| No | 40 (53.3%) | 35 (46.7%) | 0.0547^3^ |
| Yes | 0 (0.0%) | 4 (100.0%) |  |
| Total | 40 | 39 |  |
| **Chronic obstructive pulmonary disease (N, %)** |  |  |  |
| No | 40 (52.6%) | 36 (47.4%) | 0.1156^3^ |
| Yes | 0 (0.0%) | 3 (100.0%) |  |
| Total | 40 | 39 |  |

SD, standard deviation; N, sample number; ^1^: based on Mann-Whitney test; ^2^, Chi-square test; ^3^, Fisher's exact test.

**Table S3.** Fold regulation and p value for each validated miRNA.

| **miRNA** | **Fold regulation** | **p-value*** |
| --- | --- | --- |
| *Normalized by hsa-miR-34a-3p* |  |  |
| Has-miR-4433b-5p | -1,12 | 0.2826 |
| Has-miR-320b | 1.91 | **0.0090** |
| Has-miR-16-2-3p | 1.84 | 0.2630 |
| *Normalized by hsa-miR-205-3p* |  |  |
| Has-miR-4433b-5p | 5.39 | 0.4001 |
| Has-miR-320b | 19.81 | **0.0424** |
| Has-miR-16-2-3p | 11.50 | 0.6490 |

* based on Mann-Whitney test.

**Table S4.** TargetScan predicted gene interactions for hsa-miR-320b.

| **#** | **Gene name** | **Description** | **Function** |
| --- | --- | --- | --- |
| 1 | SLC9A2 | Human Na+/H+ exchanger | The Na^+^/H^+^exchangers belong to a family of transport proteins involved in intracellular pH regulation and vectorial sodium transport across various epithelial tissues [1]. |
| 2 | RPA3OS | Heterotrimeric replication protein A complex (RPA) | RPA3 is a part of RPA, which plays an essential role in DNA replication and the cellular response to DNA damage. RPA has also been reported to regulate telomere elongation through modulation of telomerase activity [2]. |
| 3 | CYP1A2 | Cytochrome P450 enzyme | CYP1A2 is one of the most important cytochrome P450 (CYP) enzymes in the liver that metabolises many clinical drugs [3]. |
| 4 | ADAL | Adenosine deaminase-related genes | The members of Adenosine Deaminase (ADA) family, including the similar protein grouped named ADAL (adenosine deaminase “like”) are responsible for cleaving the neuromodulator adenosine to inosine [4]. |
| 5 | LPPR1 | Plasticity-related gene 3 | *LPPR1* is one of the 5 members of a brain-specific gene family that modulates neuronal plasticity during development, aging, and after brain injury [5]. |
| 6 | BHLHE41 | The circadian rhythm-related genes | Circadian rhythm-related genes, such as BHLHE41/DEC2, have various functions under different cellular and tissue conditions, having been reported as both a cancer suppressor gene and an oncogenic gene [6]. |
| 7 | HSPH1 | Heat shock protein | Heat shock proteins are involved in ferroptosis [7]. |
| 8 | VEPH1 | Ventricular Zone Expressed PH Domain Containing 1 | VEPH1 is an adaptor protein capable of modulating multiple signal transduction networks [8]. |
| 9 | ARL8B | Arf-like GTP-binding protein | Arl8b regulates cargo trafficking and positioning of lysosomes [9]. |
| 10 | PBX3 | Pre-B cell leukemia (PBX) family member | PBX3 is frequently associated with cancer, where it has a role in promoting cell survival, invasion, and proliferation [10]. |
| 11 | MLF1 | Human leukemia-associated myeloid leukemia factor 1 | MLF1 is associated with human cancer progression [11]. |
| 12 | KITLG | Stem cell factor | *KITLG* is a ligand of the KIT tyrosine kinase receptor, and it is a major growth factor of mast cells [12]. |
| 13 | DNER | Non-canonical Notch ligand | DNER was first described to be highly expressed by Purkinje neurons where it played a key role in cerebellum development, but it is also involved in differentiation and proliferation during cancer and stemness [13]. |
| 14 | CDK13 | Transcriptional Cyclin Dependent Kinases | CDKs are a family of kinases activated by a cyclin binding partner that have roles in controlling transcriptional subprocesses including initiation, elongation, co-transcriptional RNA processing, and termination [14]. |
| 15 | ARFIP1 | ADP-ribosylation factor interacting protein 1 | ADP-ribosylation factors (Arfs) and Arf-like (Arl) GTPases are key regulators of intracellular vesicle trafficking and Golgi structure [15]. |
| 16 | ST7-OT4 | ST7 overlapping transcript 4 | ST7-OT4 is a non-conding RNA [16]. |
| 17 | CD3G | T-cell receptor/CD3 (TCR/CD3) complex member | The T cell receptor/CD3 (TCR/CD3) complex is crucial for T cell maturation and is associated with the immune response [17]. |
| 18 | CD12orf36 | - | - |
| 19 | FAM63B | Family with seq-uence similarity 63, member B | The biological function of *FAM63B* is currently not well described. The gene is expressed across most tissues, with highest expression in the cerebellar hemisphere and cerebellum [18]. |
| 20 | RGS9BP | Regulator of G-protein signaling 9 (RGS9) GTPase-accelerating protein (GAP) complex | Rgs9bp encodes R9AP, the anchor protein of the Rgs9 GAP photoreceptor complex, which is associated with the regulation of visual signaling [19]. |
| 21 | GCG | Preproglucagon gene | The preproglucagon gene (Gcg) encodes multiple peptides including glucagon, glucagon-like peptide-1, glucagon-like peptide-2, oxyntomodulin, and glicentin. Of these, glucagon and GLP-1 have received the most attention because of important roles in glucose metabolismo [20]. |

References:

[1] Ghishan, F.K.; Knobel, S.M.; Summar, M. Molecular Cloning, Sequencing, Chromosomal Localization, and Tissue Distribution of the Human Na+/H+exchanger (SLC9A2). *Genomics* **1995**, *30*, 25–30, doi:10.1006/geno.1995.0004.

[2] Shirai, Y.; Honda, S.; Ikari, K.; Kanai, M.; Takeda, Y.; Kamatani, Y.; Morisaki, T.; Tanaka, E.; Kumanogoh, A.; Harigai, M.; et al. Association of the RPA3-UMAD1 Locus with Interstitial Lung Diseases Complicated with Rheumatoid Arthritis in Japanese. *Ann. Rheum. Dis.* **2020**, *79*, 1305–1309, doi:10.1136/annrheumdis-2020-217256.

[3] Guo, J.; Zhu, X.; Badawy, S.; Ihsan, A.; Liu, Z.; Xie, C.; Wang, X. Metabolism and Mechanism of Human Cytochrome P450 Enzyme 1A2. *Curr. Drug Metab.* **2021**, *22*, 40–49, doi:10.2174/1389200221999210101233135.

[4] Rosemberg, D.B.; Rico, E.P.; Guidoti, M.R.; Dias, R.D.; Souza, D.O.; Bonan, C.D.; Bogo, M.R. Adenosine Deaminase-Related Genes: Molecular Identification, Tissue Expression Pattern and Truncated Alternative Splice Isoform in Adult Zebrafish (Danio Rerio). *Life Sci.* **2007**, *81*, 1526–1534, doi:10.1016/j.lfs.2007.09.019.

[5] Wallen, Z.D.; Chen, H.; Hill-Burns, E.M.; Factor, S.A.; Zabetian, C.P.; Payami, H. Plasticity-Related Gene 3 ( LPPR1 ) and Age at Diagnosis of Parkinson Disease . *Neurol. Genet.* **2018**, *4*, e271, doi:10.1212/nxg.0000000000000271.

[6] Furukawa, T.; Mimami, K.; Nagata, T.; Yamamoto, M.; Sato, M.; Tanimoto, A. Approach to Functions of BHLHE41/DEC2 in Non-Small Lung Cancer Development. *Int. J. Mol. Sci.* **2023**, *24*, 1–12, doi:10.3390/ijms241411731.

[7] Xu, X.; Li, Y.; Wu, Y.; Wang, M.; Lu, Y.; Fang, Z.; Wang, H.; Li, Y. Increased ATF2 Expression Predicts Poor Prognosis and Inhibits Sorafenib-Induced Ferroptosis in Gastric Cancer. *Redox Biol.* **2023**, *59*, 102564, doi:10.1016/j.redox.2022.102564.

[8] Brown, T.J.; Kollara, A.; Shathasivam, P.; Ringuette, M.J. Ventricular Zone Expressed PH Domain Containing 1 (VEPH1): An Adaptor Protein Capable of Modulating Multiple Signaling Transduction Pathways during Normal and Pathological Development. *Cell Commun. Signal.* **2019**, *17*, 1–13, doi:10.1186/s12964-019-0433-4.

[9] Rawat, S.; Chatterjee, D.; Marwaha, R.; Charak, G.; Kumar, G.; Shaw, S.; Khatter, D.; Sharma, S.; de Heus, C.; Liv, N.; et al. RUFY1 Binds Arl8b and Mediates Endosome-to-TGN CI-M6PR Retrieval for Cargo Sorting to Lysosomes. *J. Cell Biol.* **2023**, *222*, doi:10.1083/jcb.202108001.

[10] Morgan, R.; Pandha, H.S. PBX3 in Cancer. *Cancers (Basel).* **2020**, *12*, 1–11, doi:10.3390/cancers12020431.

[11] Tang, Z. Epigenetic Deregulation of MLF1 Drives Intrahepatic Cholangiocarcinoma Progression through EGFR / AKT and Wnt / β -Catenin Signaling. **2023**, 1–18, doi:10.1097/HC9.0000000000000204.

[12] Mak, A.C.Y.; Sajuthi, S.; Joo, J.; Xiao, S.; Sleiman, P.M.; Lurmann, F.; Jain, D.; Abecasis, G.; Kang, H.M.; Nickerson, D.A.; et al. Lung Function in African American Children with Asthma Is Associated with Novel Regulatory. *215*, 869–886.

[13] Ballester-López, C.; Conlon, T.M.; Ertüz, Z.; Greiffo, F.R.; Irmler, M.; Verleden, S.E.; Beckers, J.; Fernandez, I.E.; Eickelberg, O.; Yildirim, A.Ö. The Notch Ligand DNER Regulates Macrophage IFNγ Release in Chronic Obstructive Pulmonary Disease. *EBioMedicine* **2019**, *43*, 562–575, doi:10.1016/j.ebiom.2019.03.054.

[14] Insco, M.L.; Abraham, B.J.; Dubbury, S.J.; Kaltheuner, I.H.; Dust, S.; Wu, C.; Chen, K.Y.; Liu, D.; Bellaousov, S.; Cox, A.M.; et al. Oncogenic CDK13 Mutations Impede Nuclear RNA Surveillance. *Science* **2023**, *380*, eabn7625, doi:10.1126/science.abn7625.

[15] Feng, H.P.; Cheng, H.Y.; Hsiao, T.F.; Lin, T.W.; Hsu, J.W.; Huang, L.H.; Yu, C.J. ArfGAP1 Acts as a GTPase-Activating Protein for Human ADP-Ribosylation Factor-like 1 Protein. *FASEB J.* **2021**, *35*, doi:10.1096/fj.202000818RR.

[16] Vincent, J.B.; Petek, E.; Thevarkunnel, S.; Kolozsvari, D.; Cheung, J.; Patel, M.; Scherer, S.W. The RAY1/ST7 Tumor-Suppressor Locus on Chromosome 7q31 Represents a Complex Multi-Transcript System. *Genomics* **2002**, *80*, 283–294, doi:10.1006/geno.2002.6835.

[17] Rowe, J.H.; Delmonte, O.M.; Keles, S.; Stadinski, B.D.; Dobbs, A.K.; Henderson, L.A.; Yamazaki, Y.; Allende, L.M.; Bonilla, F.A.; Gonzalez-Granado, L.I.; et al. Patients with CD3G Mutations Reveal a Role for Human CD3g in Treg Diversity and Suppressive Function. *Blood* **2018**, *131*, 2335–2344, doi:10.1182/blood-2018-02-835561.

[18] Starnawska, A.; Demontis, D.; McQuillin, A.; O’Brien, N.L.; Staunstrup, N.H.; Mors, O.; Nielsen, A.L.; Børglum, A.D.; Nyegaard, M. Hypomethylation of FAM63B in Bipolar Disorder Patients. *Clin. Epigenetics* **2016**, *8*, 1–6, doi:10.1186/s13148-016-0221-6.

[19] Sundermeier, T.R.; Vinberg, F.; Mustafi, D.; Bai, X.; Kefalov, V.J.; Palczewski, K. R9AP Overexpression Alters Phototransduction Kinetics in ICre75 Mice. *Investig. Ophthalmol. Vis. Sci.* **2014**, *55*, 1339–1347, doi:10.1167/iovs.13-13564.

[20] Sandoval, D.A.; D’Alessio, D.A. Physiology of Proglucagon Peptides: Role Ofglucagon and GLP-1 in Health and Disease. *Physiol. Rev.* **2015**, *95*, 513–548, doi:10.1152/physrev.00013.2014.
